# Comprehensive chemical, morphological, thermal, and biological characterization of *Agave tequilana* extract and chitosan-based dissolving microneedle arrays

**DOI:** 10.1371/journal.pone.0350922

**Published:** 2026-06-05

**Authors:** Patricia Quintero-Rincón, Karina Caballero-Gallardo, Oscar Flórez-Acosta

**Affiliations:** 1 Research Group Design and Formulation of Medicines, Cosmetics, and Related, Faculty of Pharmaceutical and Food Sciences, Universidad de Antioquia, Medellin, Colombia; 2 Functional Toxicology Group, School of Pharmaceutical Sciences, Zaragocilla Campus, University of Cartagena, Cartagena, Colombia; Siksha O Anusandhan University School of Pharmaceutical Sciences, INDIA

## Abstract

Dissolving microneedles have gained attention as minimally invasive platforms for transdermal research applications. In this study, chitosan‑based dissolving microneedle arrays incorporating a hydroalcoholic extract of *Agave tequilana* root were formulated and systematically characterized. Chemical profiling by UHPLC-ESI-Orbitrap-MS revealed a diverse phytochemical composition dominated by phenolic acids, particularly rosmarinic acid. Morphological and structural analyses showed that extract incorporation induced concentration‑dependent changes in microneedle geometry: arrays containing 0.25% extract preserved tip sharpness, whereas a 0.50% loading led to surface roughening and partial tip deformation. Thermal analyses demonstrated extract‑dependent modifications in material degradation behavior, consistent with interactions between chitosan and extract constituents. Biological assays confirmed that the agave extract exhibits high antioxidant capacity, moderate tyrosinase inhibition, and inhibitory activity against collagenase and hyaluronidase. Following incorporation into the microneedle arrays, antioxidant activity and inhibitory effects against tyrosinase and hyaluronidase were retained, while collagenase inhibition was reduced, particularly in the 0.25% formulation. In vitro cytotoxicity assays indicated biocompatibility toward HaCaT keratinocytes and concentration-dependent selective cytotoxicity in A375 melanoma cells. Taken together, these findings indicate that extract loading is a critical parameter influencing structural preservation and *in vitro* bioactivity of chitosan‑based dissolving microneedles. Further studies addressing mechanical performance, insertion behavior, matrix dissolution, and skin permeation are required to evaluate the functional reliability of this system.

## Introduction

Microneedles (MN) are a minimally invasive transdermal delivery platform that enhance skin permeation by creating transient microchannels across the stratum corneum, thereby improving bioavailability while avoiding first-pass metabolism and tissue damage [1,2]. Among MN designs, dissolving microneedles (DMN) are of particular interest because they encapsulate bioactive agents within biodegradable matrices that dissolve after insertion, enabling safe and localized administration without sharp waste [3–7]. DMN systems have been investigated for multiple dermatological and therapeutic applications [8–19], although their performance depends strongly on material properties. In this regard, chitosan has emerged as a suitable DMN-forming polymer due to its biocompatibility, biodegradability, mechanical integrity, and ability to interact with bioactive compounds and skin tissues [20–25].

Plant-derived extracts constitute a rich source of bioactive compounds with antioxidant, anti-inflammatory and antiproliferative activities relevant to skin applications [26,27]. Although their incorporation into polymer-based microneedle systems is attracting increasing interest, systematic evaluations of extract-DMN interactions remain scarce, particularly regarding physicochemical stability and biological safety. This limitation is notable for underexplored medicinal species with dermocosmetic potential. *Agave tequilana* F.A.C.Weber var. azul (Asparagaceae), commonly known as agave, maguey, fique or cabuya, exhibits a diverse phytochemical profile, including fructans, phenolic acids and saponins associated with ant ioxidant, immunomodulatory, antibacterial and hypoglycemic effects [28–37], and has been proposed as a functional ingredient for dermocosmetic and biomaterial applications [38]. However, its integration into chitosan-based DMN platforms and the resulting combined properties remain insufficiently characterized.

Based on these considerations, we propose to develop and characterize chitosan-based DMN arrays loaded with *A. tequilana* root extract as a biologically safe platform capable of exhibiting antioxidant activity and inhibitory effects on skin-relevant enzymes, without compromising transdermal delivery performance. In this context, the main objective of this study is to perform a systematic characterization of the agave extract and DMN arrays, both loaded and non-loaded with the extract, from a physicochemical, thermal, and biological perspective, thus addressing the current lack of integrated evidence supporting these hybrid systems.

To this end, the chemical profile of the agave root extract was identified using UHPLC-ESI-ORBITRAP-MS, while the morphological, elemental, and thermal properties of the raw materials and DMN arrays were evaluated using SEM, EDS, TGA, and DSC. Antioxidant capacity and enzyme inhibition assays relevant to skin physiology were performed, and biological safety was assessed using HaCaT keratinocytes, along with anti-melanoma activity in A375 cells. This study presents an evidence-based evaluation of chitosan-agave DMN arrays as potential dermocosmetic platforms, explicitly acknowledging their experimental scope and current limitations, and providing crucial information for their future validation and optimization.

## Results

### Chemical, morphological and thermal characterization

#### Chemical analysis.

The chemical profile of the *A. tequilana* root extract was analyzed by UHPLC-ESI-ORBITRAP-MS, allowing the identification and quantification of twenty phytocompounds (Table 1). Three compounds were classified as alkaloids (theobromine, theophylline, and caffeine), nine as flavonoids (epigallocatechin gallate, epicatechin, epicatechin gallate, rutin, luteolin, quercetin, naringenin, apigenin, and pinocembrin), seven as phenolic acids (*p*-hydroxybenzoic, caffeic, vanillic, *p*-coumaric, ferulic, rosmarinic, and *trans*‑cinnamic acids), and one compound as a pentacyclic triterpenoid (ursolic acid). Among the identified constituents, rosmarinic acid was the most abundant, with a concentration of 69.6 mg/kg of dry sample, followed by *p*-hydroxybenzoic acid (6.5 mg/kg) and *p*-coumaric acid (1.0 mg/kg).

**Table 1:** Identification and quantification of phytocompounds in the *A. tequilana* extract by UHPLC-ESI-ORBITRAP-MS.

No.	Compound	T_R_^a^, min	MQL ^b^, mg/kg	Concentration, mg/kg of dry sample
1	Theobromine	2.9	0.4	<0.4
2	Theophylline	3.4	0.4	<0.4
3	*p*-Hydroxybenzoic acid	3.4	0.4	**6.5**
4	Caffeine	3.8	0.4	<0.4
5	Caffeic acid	3.9	0.4	0.4
6	Epigallocatechin gallate	3.9	0.4	<0.4
7	Epicatechin	4.0	0.4	<0.4
8	Vanillic acid	4.3	0.4	<0.4
9	*p*-Coumaric acid	4.5	0.4	1.0
10	Epicatechin gallate	4.5	0.4	<0.4
11	Ferulic acid	5.2	0.4	<0.4
12	Rosmarinic acid	5.2	10.0	**69.6**
13	Rutin	5.2	0.4	<0.4
14	*trans*-Cinnamic acid	5.9	2.0	<2.0
15	Quercetin	6.0	0.4	<0.4
16	Naringenin	6.0	0.4	<0.4
17	Luteolin	6.2	0.4	<0.4
18	Apigenin	6.5	0.4	<0.4
19	Pinocembrin	6.9	0.4	<0.4
20	Ursolic acid	9.3	0.4	<0.4

^a^Retention time (T_R_). ^b^Minimum quantification level (MQL).

### Morphological and elemental analysis of *A. tequilana* extract and chitosan

**Fig 1:**
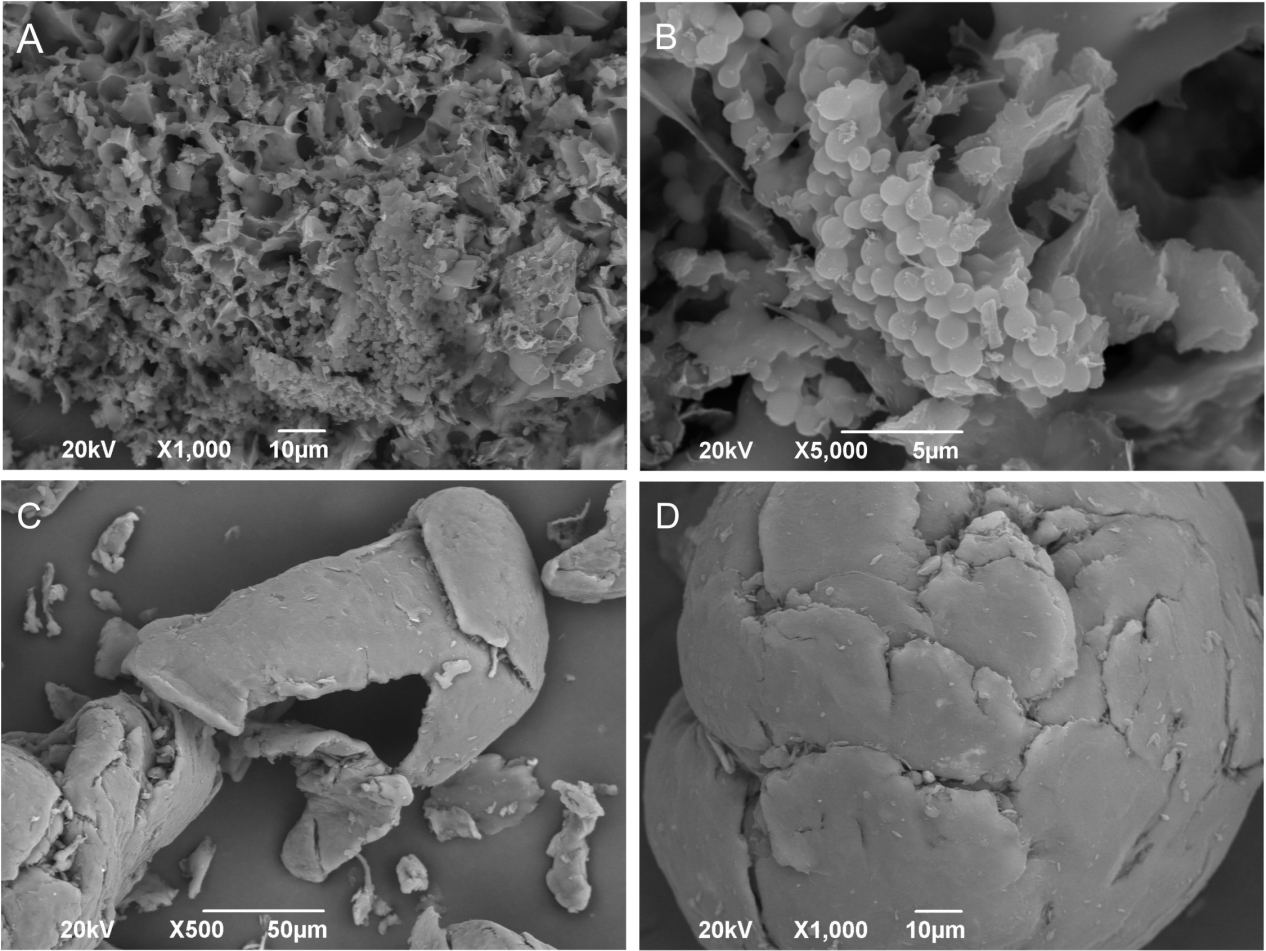
SEM micrographs of *A. tequilana* extract (A, B) and chitosan (C, D).

The morphological features of the *A. tequilana* extract and chitosan were examined by optical microscopy (Fig 1). As shown in Fig 1A, the extract exhibited a heterogeneous microstructure characterized by a porous matrix with irregular, interconnected cavities and a rough surface. Lamellar fragments and amorphous aggregates were distributed throughout the matrix. At higher magnification (5 µm scale; Fig 1B), the extract displayed a laminar background with dispersed spherical particles of approximately 2 µm in diameter.

Chitosan micrographs (Fig 1C–1D), obtained at 500× and 1000 × magnification, showed an irregular but comparatively compact morphology. Large lamellar fragments with smooth surfaces and well‑defined edges were observed. The material appeared largely homogeneous, with compact regions and surface fractures visible in some areas. No porous structures or particulate aggregates were detected under the conditions analyzed.

Elemental composition of the agave extract and chitosan was analyzed by energy-dispersive X-ray spectroscopy (EDS) (S1–S2 Figs; S1–S2 Tables). The EDS spectra of the agave extract showed dominant signals corresponding to carbon (C) and oxygen (O). Potassium (K) was detected at low intensity. No additional elements were detected within the sensitivity limits of the technique.

Similarly, EDS spectra of chitosan were dominated by carbon and oxygen signals, with no detectable peaks corresponding to metallic elements or inorganic residues. Nitrogen (N) and hydrogen (H) were not detected in either material, consistent with the known limitations of EDS for light elements.

#### Morphological and structural analysis of DMN arrays.

**Fig 2:**
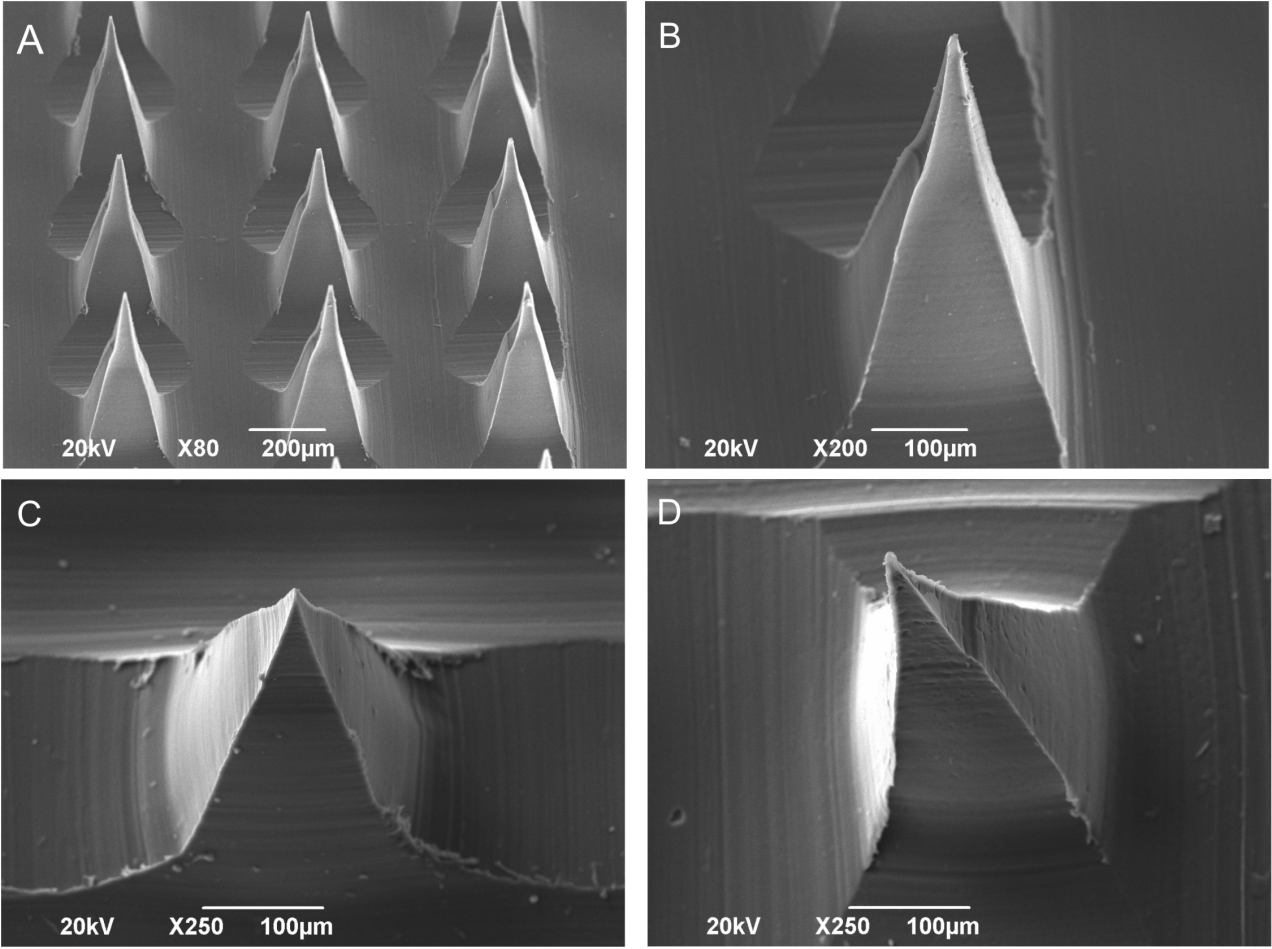
SEM micrographs of the non-loaded DMN (A, B) and extract-loaded DMN: *A. tequilana* extract to 0.25% (C), and *A. tequilana* extract to 0.50% (D).

Fig 2 presents SEM micrographs of chitosan-based DMN arrays under two conditions: non‑loaded DMN (Fig 2A–2B) and DMN loaded with *A. tequilana* extract at concentrations of 0.25% (Fig 2C) and 0.50% (Fig 2D).

The non-loaded DMN arrays (Fig 2A) exhibited a regular pyramidal geometry, with microneedles uniformly distributed on the substrate. The microneedle walls appeared smooth, and the edges and tips were well defined. A higher-magnification image of an individual non-loaded microneedle (Fig 2B) showed a sharp tip and smooth lateral surfaces, with linear features associated with the molding process. No collapsed structures or visible structural discontinuities were observed.

In DMN loaded with 0.25% extract (Fig 2C), the overall morphology was preserved; however, slight roughness appeared on the lateral surfaces, with reduced sharpness at the tip and the presence of burrs or residues at the base, attributed to changes in viscosity during extract incorporation. At 0.50% extract loading (Fig 2D), more pronounced morphological alterations were observed, including tip blunting, increased wall roughness, and partial structural collapse, suggesting significant changes in morphological preservation and structural appearance at higher extract concentrations.

#### Thermogravimetric analysis.

**Fig 3:**
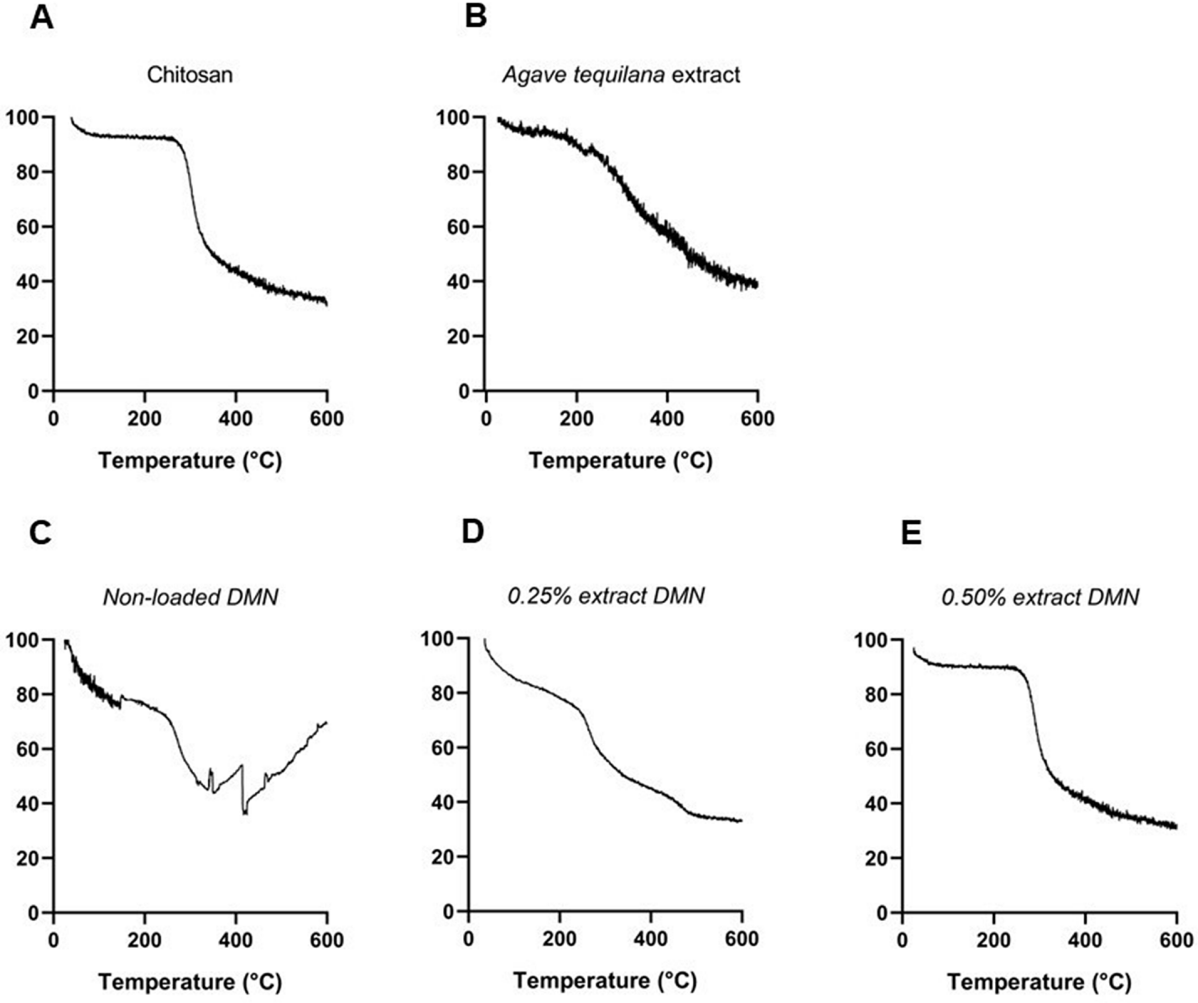
TGA thermogram of *A. tequilana* extract (A), chitosan (B), non-loaded CS-DMN (C), and CS-DMN loaded with extract: 0.25% *A. tequilana* extract (D) and 0.50% *A. tequilana* extract (E).

The thermal behavior of chitosan, *A. tequilana* extract, and chitosan‑based DMN arrays, both non‑loaded and extract‑loaded, was analyzed by thermogravimetric analysis (TGA) under a nitrogen atmosphere (Fig 3). Differential thermal analysis (DTA) profiles are shown in S3 Fig.

Commercial chitosan (Fig 3A) exhibited an initial mass loss in the temperature range of 50–100 °C. A second, more pronounced mass-loss event was observed between 270 and 350 °C. At 600 °C, the remaining residue accounted for approximately 30% of the initial mass.

The *A. tequilana* extract (Fig 3B) showed a gradual mass loss starting at approximately 50 °C, followed by a continuous decomposition process extending up to 400 °C. No single, sharply defined degradation step was observed. The residual mass at 600 °C was also approximately 30%.

Non‑loaded DMN arrays (Fig 3C) exhibited a multistep mass-loss profile. An initial mass loss was observed around 100 °C, followed by a main degradation event between 270 and 350 °C. Additional mass changes were detected in the range of 350–500 °C. The residual mass at 600 °C was comparable to that observed for chitosan.

DMN loaded with 0.25% *A. tequilana* extract (Fig 3D) showed an onset of mass loss at approximately 200 °C, followed by a continuous decomposition process extending up to 450 °C. Compared with non-loaded DMN, the degradation transitions appeared broader. The residual mass at 600 °C was approximately 35%.

At an extract loading of 0.50% (Fig 3E), the DMN arrays exhibited an initial mass loss around 100 °C. The main decomposition occurred primarily between 200 and 400 °C, with no sharply defined single degradation step. The residual mass at 600 °C ranged between 30 and 35%.

DTA profiles are shown in S3 Fig. Chitosan (A) displayed a prominent endothermic event in the temperature range of 300–400 °C, preceded by smaller thermal events between 70 and 120 °C. The *A. tequilana* extract (B) exhibited broad, low‑intensity thermal signals distributed over a wide temperature range.

The DTA profile of non‑loaded DMN (C) showed a main thermal event centered near 350 °C with lower intensity compared to chitosan. DMN loaded with 0.25% extract (S3D Fig) exhibited a slightly shifted main peak with reduced intensity. At 0.50% extract loading (D), multiple overlapping thermal events were observed, with increased signal intensity between 300 and 400 °C.

### Differential scanning calorimetry

**Fig 4:**
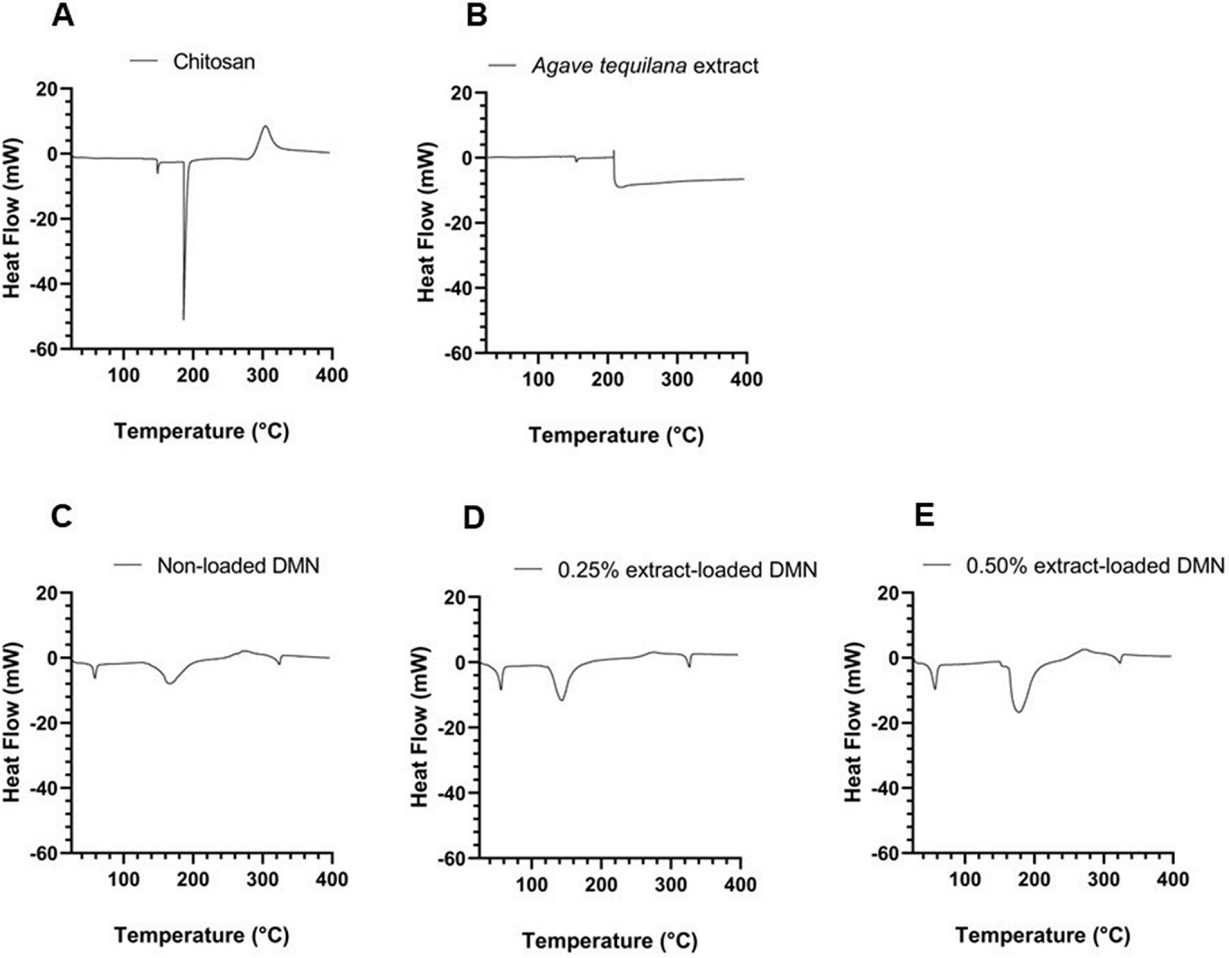
DSC thermogram of chitosan (A), *A. tequilana* extract (B), non-loaded DMN (C), and DMN loaded with extract: 0.25% *A. tequilana* extract (D) and 0.50% *A. tequilana* extract (E).

The differential scanning calorimetry (DSC) thermogram of commercial chitosan (Fig 4A) showed an endothermic event centered around 200 °C. No additional thermal events were detected within the analyzed temperature range.

The thermogram of the *A. tequilana* extract (Fig 4B) exhibited an endothermic transition in a similar temperature region, with a broader profile compared to chitosan. No discrete, well‑defined additional peaks were observed across the scanned range.

For the non‑loaded DMN arrays (Fig 4C), the DSC thermogram showed no pronounced thermal events within the temperature range of 100–400 °C. No additional transitions were detected in this interval.

In contrast, the thermogram of DMN loaded with 0.25% *A. tequilana* extract (Fig 4D) exhibited a modified thermal profile compared with the non-loaded system. A shift in the main thermal transition and a lower peak intensity were observed.

At an extract loading of 0.50% (Fig 4E), the thermogram displayed further deviations relative to the non‑loaded DMN, including changes in peak shape and the appearance of additional thermal features across the analyzed range.

### Biological characterization

#### Antioxidant capacity.

The antioxidant activity of the *A. tequilana* extract and the DMN formulations was evaluated using FRAP and ABTS assays (Table 2). The *A. tequilana* extract showed FRAP values of 42,549.8 µmol TE/100 g and ABTS values of 23,940.7 µmol TE/100 g.

**Table 2:** Antioxidant capacity of *A. tequilana* extract and DMN arrays.

Sample	TEAC (µmol TE/100 g sample or DMN)	
	FRAP	TEAC-ABTS
*Extract of Agave tequilana* roots	42,549.8 ± 147.7	23,940.7 ± 134.9
Non-loaded DMN	302.9 ± 19.8	2.5 ± 0.2
0.25% extract-loaded DMN	2,214.9 ± 114.8	376.8 ± 23.9
0.50% extract-loaded DMN	1,237.6 ± 7.7	197.2 ± 2.6

TEAC (Trolox equivalent antioxidant capacity). FRAP (Ferric ion reducing antioxidant power). ABTS (2,2’-azinobis (3-ethylbenzothiazoline-6-sulfonic acid, diammonium salt). DMN (microneedles).

For the DMN arrays, antioxidant activity varied according to extract loading. DMN loaded with 0.25% extract exhibited FRAP values of 2,214.9 µmol TE/100 g of DMN and ABTS values of 376.8 µmol TE/100 g of DMN. DMN loaded with 0.50% extract showed lower values, with FRAP and ABTS values of 1,237.6 and 197.2 µmol TE/100 g of DMN, respectively. In contrast, non‑loaded DMN exhibited FRAP values of 302.9 µmol TE/100 g of DMN and ABTS values of 2.5 µmol TE/100 g of DMN.

#### Enzymatic inhibitory activity.

Table 3 summarizes the inhibitory activity of the *A. tequilana* extract and the DMN formulations against tyrosinase, collagenase, and hyaluronidase. The *A. tequilana* extract showed tyrosinase inhibition of 21.9 ± 2.7%, while DMN loaded with 0.25% extract exhibited an inhibition of 26.6 ± 0.9%. Non‑loaded DMN showed lower levels of inhibition under the tested conditions.

**Table 3:** Enzymatic inhibitory activity on tyrosinase, collagenase, and hyaluronidase of *A. tequilana* extract and DMN arrays.

Sample	Enzyme inhibition (%)		
	Tyrosinase	Collagenase	Hyaluronidase
*Extract of Agave tequilana* roots, 250 µg/mL	21.9 ± 2.7	66.3 ± 2.4	76.2 ± 3.0
Non-loaded DMN	2.9 ± 0.1	5.0 ± 1.2	18.5 ± 0.9
0.25% extract-loaded DMN	26.6 ± 0.9	13.4 ± 1.2	21.7 ± 0.5
0.50% extract-loaded DMN	3.9 ± 0.9	14.6 ± 2.1	5.2 ± 0.2
Kojic acid, 100 µM	99.0 ± 0.1	ND	ND
Epigallocatechin gallate, 5 mM	ND	91.4 ± 1.1	ND
Epigallocatechin gallate, 10 mM	ND	ND	93.8 ± 1.4

ND (Not determined). DMN (microneedles).

For collagenase, the extract exhibited an inhibition of 66.3 ± 2.4%. In contrast, both extract-loaded and non-loaded DMN arrays displayed limited inhibitory activity, with inhibition values remaining low compared to the extract.

Regarding hyaluronidase inhibition, the *A. tequilana* extract showed an inhibition of 66.3 ± 2.4%. Non-loaded DMN and 0.25% extract-loaded DMN exhibited lower inhibition values of 18.5 ± 0.9% and 21.7 ± 0.5% per patch, respectively. DMN loaded with 0.50% extract showed the lowest inhibition among the tested formulations, with a value of 5.2 ± 0.2% per patch.

#### Cytotoxicity assay.

**Fig 5:**
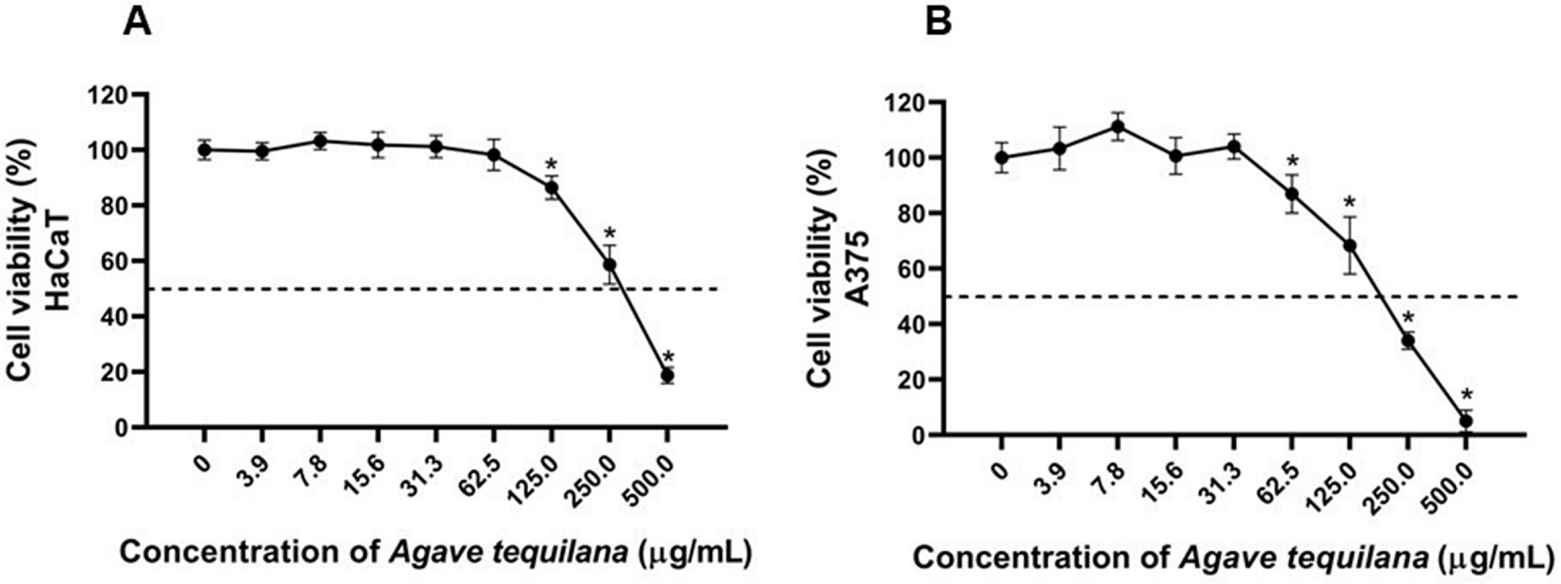
Effects of *A. tequilana* extract on viability of HaCaT cells (A) and A375 cells (B). The asterisk (*) indicates significant difference in viability compared with the group of the non-treated cells (*p* < 0.05). Data are the mean ± SEM (n = 3).

Fig 5 shows the effects of the *A. tequilana* extract on the viability of HaCaT keratinocytes (Fig 5A) and A375 melanoma cells (Fig 5B) following exposure to concentrations ranging from 0 to 500 µg/mL. In both cell lines, cell viability remained close to 100% at lower concentrations (3.9–15.6 µg/mL).

At concentrations ≥ 31.3 µg/mL, a concentration-dependent decrease in cell viability was observed, which was more pronounced in A375 cells. At the highest tested concentration (500 µg/mL), viability values in A375 cells were reduced to approximately 3%. Statistically significant differences relative to the negative control (non‑treated cells) were detected at concentrations above 125 µg/mL in both cell lines (*p* < 0.05).

The inhibitory concentration 50 (IC₅₀) values calculated for the *A. tequilana* extract were 135.9 µg/mL for A375 cells and 257.8 µg/mL for HaCaT cells (Table 4). Based on these values, a selectivity index (SI) of 1.9 was obtained.

**Table 4:** Results of IC_50_ on HaCaT and A375 cells and SI of the extract of *A. tequilana* roots.

*Extract of A. tequilana* roots	HaCaT	A375	SI	Observation
**IC**_**50**_ **(µg/mL)**	257.8	135.9	1.9	Desirable selectivity
**95% CI**	158.3 to 419.9	109.4 to 168.3		
**R** ^**2**^	0.7363	0.8199		

IC_50_ (inhibitory concentration 50). CI (confidence interval). SI (selectivity index).

**Fig 6:**
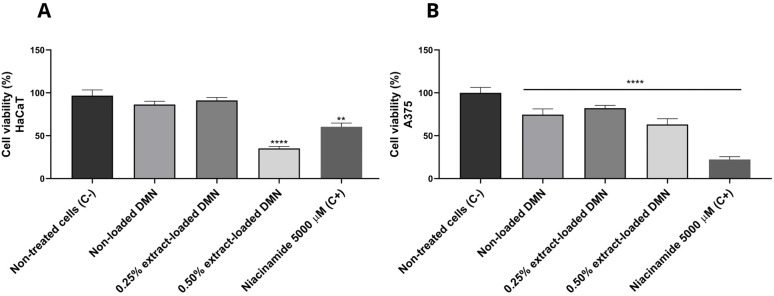
Effects of non-loaded DMN, 0.25% *A. tequilana* extract-loaded DMN and 0.50% *A. tequilana* extract-loaded DMN on HaCaT cells (A) and A375 cells (B). The asterisk (*) indicates significant difference in viability compared with the group of the non-treated cells (*p* < 0.05). Data are the mean ± SEM (n = 3).

The results of the cytotoxic activity of DMN arrays shown in Fig 6 indicate that the chitosan DMN, both non-loaded and loaded with 0.25% *A. tequilana* root extract, did not present a significant reduction in the cell viability of the HaCaT cells (Fig 6A) compared to the negative control (100% viability), and evidencing biocompatibility. In addition, a decrease in cell viability was observed for the DMN loaded with 0.50% extract, being statistically significant for HaCaT (Fig 6A) and A375 (Fig 6B). The non-loaded DMN and those loaded with 0.25% extract showed a modest but significant reduction in the viability of A375. The positive control with 5,000 μM niacinamide showed a significant and marked reduction in cell viability in both lines (approximately 35% for HaCaT and 20% for A375), validating the assay’s sensitivity.

## Discussion

This study contributes to the development of chitosan‑based DMN arrays incorporating a hydroalcoholic extract of agave root, with emphasis on examining how the extract’s chemical composition influences formulation‑level attributes such as extract incorporation within the polymeric matrix, distribution homogeneity, and preservation of microneedle geometry. Rather than establishing transdermal performance, the present work focuses on structural and compositional relationships relevant to DMN fabrication. Dissolving microneedles are widely explored as minimally invasive systems for skin‑interfacing applications, and their formulation with chitosan provides a biocompatible, biodegradable, low‑toxicity polycationic matrix. The presence of amino and hydroxyl functional groups confers physicochemical versatility to chitosan, enabling non‑covalent interactions with bioactive compounds within the polymer network [20,39,40].

Analysis of the chemical profile of the agave extract revealed a complex phytochemical composition dominated by phenolic acids (Table 1), consistent with reports describing the high abundance of this class of metabolites in plants adapted to arid and semi-arid ecosystems [41,42]. Rosmarinic acid was identified as the major component, supporting the extract’s reported antioxidant and anti‑inflammatory relevance [43]. However, the nominal concentration of extract incorporated into the DMN matrices cannot be directly equated with an effective dermal dose, as bioavailability depends on multiple system-related factors, including the amount of extract per microneedle, matrix dissolution behavior, and release and permeation processes within cutaneous microenvironments. Because these factors collectively determine the bioavailable fraction of the extract, their systematic evaluation should be addressed in future studies to define the actual transdermal performance of the formulated system.

From a mechanistic standpoint, rosmarinic acid has been associated with attenuation of oxidative stress-induced damage and modulation of inflammatory signaling pathways, including MAPK- and NF-κB‑related cascades, across diverse preclinical models. Protective effects on keratinocytes exposed to environmental stressors have also been reported [43,44]. These mechanisms provide a plausible rationale for further investigating the incorporation of rosmarinic acid into dermatological formulations, while emphasizing the need to validate dose-response relationships within the final formulation [45].

In addition to rosmarinic acid, other phenolic acids identified in the extract, such as *p*-hydroxybenzoic acid and *p*-coumaric acid, together with additional secondary metabolites, may contribute complementary antioxidant, antimicrobial, and anti-inflammatory activities [38]. Although synergistic interactions among these compounds are conceivable, such effects were not evaluated in the present study and should be confirmed through targeted experimental approaches.

It is important to note that these phytochemicals are unlikely to be incorporated inertly within the polymeric matrix. Polyphenols can interact with polysaccharides through hydrogen bonding, electrostatic interactions, and hydrophobic forces [46,47]. In chitosan-based DMN systems, such interactions may function as non-covalent crosslinking points, thereby modifying polymer network packing and influencing microneedle morphology and thermal behavior [46]. However, the extent and functional relevance of these interactions were not directly assessed in this study.

The properties associated with the compounds identified in the agave extract support its incorporation into chitosan-based DMN arrays as a strategy to preserve measurable bioactivity within a polymeric matrix. In contrast to previously reported multicomponent microneedle formulations combining saponin extracts from *Panax notoginseng* (Burkill) F.H.Chen, rhodioloside, and madecassoside to induce synergistic rejuvenating effects [48], or microneedle platforms loaded with asiatic acid derived from *Centella asiatica* (L.) Urb. [49], the use of a single agave extract allows a more direct and controlled assessment of formulation‑dependent biological responses. Similarly, systems incorporating curcumin into polysaccharide matrices derived from *Codonopsis pilosula* (Franch.) Nannf. [50] illustrate that a central principle underlying these approaches is the selection of compatible polymeric matrices capable of maintaining the intrinsic bioactivity of plant‑derived compounds.

In this context, characterization of the individual material components provides a necessary framework for interpreting their behavior within the composite DMN system. SEM morphological analysis (Fig 1) showed that the *A. tequilana* extract exhibits a heterogeneous and highly porous structure composed of irregular cavities and lamellar fragments, resembling the honeycomb-like architectures reported for lignocellulosic byproducts [51,52]. This microstructure is consistent with the presence of cell wall remnants and polysaccharide-rich domains, which may influence interactions between the extract and the chitosan matrix. However, the morphological evidence allows only qualitative inference, rather than confirmation, that such porosity could favor the retention of bioactive compounds or affect the rheological behavior of the gel; these aspects must be addressed through dedicated dissolution and release studies [53,54]. In contrast, chitosan displayed a compact morphology with low porosity, rough surfaces, and fractured plates, consistent with its role as a structural polymer in dissolving microneedle systems [55]. Comparison of the two materials highlights the importance of controlling extract loading, as differences in microstructural features may influence the stability and structural integrity of the final patch. Elemental analysis by EDS-SEM (S1–S2 Figs; S1–S2 Tables) confirmed the absence of inorganic contaminants, supporting the chemical purity of both materials. Together, these morphological and compositional characteristics provide the material-level context necessary to interpret the structural features of the resulting microneedle arrays.

Morphological and structural characterization of microneedle arrays is critical because these features are directly related to insertion behavior, mechanical resistance, and release characteristics of incorporated bioactive compounds, which are key considerations for safe and functional transdermal system design [56,57]. Recent studies indicate that geometric parameters such as microneedle shape, tip sharpness, spacing, and density strongly influence morphological preservation and structural appearance of microneedle arrays [58]. Accordingly, preservation of these attributes is considered a central aspect in microneedle design and optimization, as it contributes to maintaining consistent structural features relevant to bioactive delivery [59].

In this study, morphological and structural analyses of the DMN arrays (Fig 2) demonstrated that incorporation of *A. tequilana* extract into chitosan matrices induced concentration-dependent modifications in surface morphology and structural preservation. Non-loaded DMN exhibited a well-defined geometry characterized by sharp tips and smooth surfaces. Incorporation of 0.25% extract resulted in mild surface roughness and minimal tip deformation, whereas a 0.50% loading led to more pronounced alterations, including tip blunting and partial structural collapse. Together, these observations suggest that increasing extract concentration alters the physical properties of the polymer matrix in a manner that may compromise microneedle structural integrity.

Taking together, these results indicate the presence of a formulation-dependent threshold in extract loading beyond which microneedle structural preservation is reduced. This finding underscores the need to balance bioactive incorporation with maintenance of structural integrity. In this context, systematic evaluation of the effects of different loading levels on DMN morphology represents an essential step toward identifying suitable formulation ranges, as reported for other microneedle-based systems [60,61].

Thermal analyses provided complementary insight into the behavior of the individual components and the formulated DMN systems. Thermogravimetric analysis indicated that commercial chitosan exhibits a well-defined degradation event between 270 and 350 °C, consistent with cleavage of the polysaccharide backbone and subsequent chain decomposition, in agreement with previously reported thermal profiles for this biopolymer [62]. In contrast, the *A. tequilana* extract displayed a broader and less sharply defined decomposition pattern, characteristic of plant-derived matrices with heterogeneous phytochemical composition. Such profiles have been attributed to the sequential degradation of fructans, simple sugars, and phenolic compounds, each contributing distinct thermal stability and staged mass-loss events [63–65]. Similar behavior has been described for agave-derived fibers and polysaccharides, underscoring the influence of compositional diversity on thermal complexity [66].

In the formulated DMN systems, extract incorporation was associated with concentration-dependent modifications in the thermal response of the chitosan matrix. Compared with non-loaded DMN, extract-loaded formulations exhibited earlier onset of degradation and broader thermal transitions, suggesting that the presence of low-molecular-weight extract constituents alters the thermal degradation pathway of the polymeric network. Comparable effects have been reported for chitosan-based systems containing phytochemicals, where non-covalent interactions between the polymer and bioactive compounds modify thermal transitions and degradation behavior [67].

Differential scanning calorimetry further supported these trends by revealing changes in enthalpy profiles and thermal event definition as a function of extract loading. In extract-loaded DMN, peak broadening, shifts in thermal transitions, and the appearance of overlapping events indicate a progressive increase in thermal complexity relative to non‑loaded systems. These observations are consistent with the superposition of thermal responses from both chitosan and extract constituents and have been widely reported in composite polysaccharide-based formulations containing heterogeneous organic phases [68].

Additionally, extract-loaded DMN formulations exhibited increased residual mass at elevated temperatures, suggesting a greater contribution of carbonaceous residues derived from the extract components. Similar increases in char formation have been described in polymer systems incorporating plant-derived materials, where aromatic and polyphenolic structures promote carbonization during thermal decomposition [67]. While the present study does not directly assess long-term thermal or storage stability, changes in thermal transitions and residue formation may influence critical parameters such as material robustness during processing or tolerance to thermal treatments, including sterilization. These aspects warrant further investigation in future studies aimed at comprehensive functional evaluation of the system [69].

From a chemical standpoint, gel matrices containing plant extracts are stabilized through a combination of physical and chemical interactions. Chitosan, as a polysaccharide, forms three-dimensional networks primarily stabilized by hydrogen bonding, ionic interactions, and physical chain entanglements [70]. Within this matrix, phenolic compounds present in the *A. tequilana* extract may further contribute by establishing additional hydrogen bonds with hydroxyl groups and, potentially, covalent interactions with reactive sites of the polymer, thereby modifying network cohesiveness and rigidity [71–73]. In parallel, polysaccharides contained in the extract may promote gelation through similar non-covalent interactions, leading to hydrated three-dimensional networks capable of retaining bioactive molecules and contributing to the structural stability of the gel matrix [74]. Additionally, proteins present in the extract may undergo denaturation during processing, generating new interaction sites, while hydrophobic interactions between aromatic moieties of phenolic compounds and non-polar regions of the matrix may further reinforce the overall structure, potentially enhancing encapsulation stability and modulating bioactive release [75]. Collectively, these interactions suggest a complex, composition‑dependent stabilization mechanism; however, their specific contributions to macroscopic properties such as mechanical integrity, dissolution behavior, and release kinetics were not evaluated in this study, limiting definitive interpretation of how these interactions translate into functional performance.

Biological assays indicated that incorporation of the *A. tequilana* extract into DMN arrays preserves measurable antioxidant, enzymatic, and selective cytotoxic activities, with responses modulated by extract concentration and extract-polymer interactions within the matrix. Antioxidant assessments confirmed that the extract exhibits strong radical‑scavenging capacity, consistent with the phenolic profile identified [76]. While DMN arrays exhibited lower antioxidant activity than the free extract, particularly at higher nominal loadings, the 0.25% formulation retained detectable activity, suggesting that a fraction of the bioactive constituents remains accessible within the polymeric network. Nevertheless, the absence of quantitative data on extract content and release limits direct interpretation of the magnitude of retained activity. Within the constraints of the current experimental scope, these observations indicate a potential contribution to mitigating oxidative stress at the formulation level [77,78].

Enzyme inhibition assays revealed moderate tyrosinase inhibition for both the extract and the 0.25% DMN arrays, consistent with reports indicating that polyphenol-enriched microneedle systems can retain functional relevance in pigmentation‑related processes [79]. Likewise, the extract exhibited inhibitory activity against collagenase and hyaluronidase, which may be associated with effects related to extracellular matrix preservation and hydration [26,80]. In contrast, the reduced inhibitory activity observed in DMN arrays at higher extract concentrations suggests that increased loading may limit enzyme accessibility or reduce immediate bioavailability, potentially due to stronger extract-polymer interactions within the matrix. This interpretation is consistent with studies demonstrating that polymer molecular weight, charge density, and microarchitecture influence both mechanical behavior and release dynamics in microneedle systems [81]. Collectively, these observations emphasize the importance of optimizing extract concentration to balance bioactive incorporation with measurable functional activity. In particular, the reduced activity detected at higher extract loadings highlights the need to incorporate quantitative release and diffusion analyses to enable a more rigorous mechanistic interpretation of bioactive accessibility within the microneedle matrix.

Cytotoxicity assays indicated that non-loaded and 0.25% extract-loaded DMN arrays were biocompatible toward HaCaT keratinocytes, while exhibiting greater cytotoxic effects in A375 melanoma cells. This differential response is consistent with the biological activity attributed to the *A. tequilana* extract when incorporated within a polymeric delivery matrix. DMN arrays loaded with 0.50% extract induced cytotoxic effects in both keratinocytes and melanoma cells, indicating the presence of a concentration-dependent threshold beyond which cellular selectivity is reduced. In contrast, non-loaded DMN and DMN formulated with lower extract loading were associated with more limited effects on keratinocyte viability, while still affecting A375 melanoma cells. The corresponding selectivity index supports a preferential sensitivity of A375 cells relative to HaCaT cells, in agreement with previous observations reported for plant-derived bioactive compounds [82,83]. However, as extract content within the DMN arrays was not quantitatively determined, these concentration-dependent effects should be interpreted cautiously, since the lack of dose quantification limits precise correlation between delivered amount and biological response.

Taken together, these findings indicate that chitosan-based dissolving microneedle arrays incorporating *A. tequilana* extract represent a versatile formulation platform for dermal and dermocosmetic research applications. While the present study establishes a foundation by elucidating formulation-level relationships between extract incorporation, matrix structure, and *in vitro* biological responses, a deeper understanding of extract-polymer interactions will be necessary to further optimize formulation stability, bioactive accessibility, and safety. In this context, several key performance parameters, including insertion efficiency, fracture resistance, skin dissolution behavior, and *ex vivo* permeation, were not evaluated and should be addressed in future investigations to achieve a more comprehensive characterization of the system. In addition, quantitative assessment of extract content uniformity using a defined chemical marker, such as rosmarinic acid, would provide critical insight into extract distribution within the patch. Incorporation of these analyses in subsequent studies will strengthen understanding of formulation behavior and support continued exploration of the translational relevance of these systems.

## Materials and methods

### Plant material

*Agave tequilana* F.A.C.Weber var. azul, voucher number DUGAND-3580, identified by Dr. Hermes Cuadros Villalobos from the “Armando Dugand Gnecco” Herbarium, University of the Atlantic, was collected in Santander de Quilichao, Cauca, Colombia. The collection site corresponds to Vereda San Antonio, characterized as an open area with grassland-like vegetation and exposed to direct solar radiation. The geographic coordinates of the site are 3° 1’ 33” N and 76° 31’ 46” W. The collection of plant material was authorized by the National Authority of Environmental Licenses (ANLA, Colombia) under Resolution No. 001579 dated July 25, 2024, and by the Regional Autonomous Corporation of the Canal del Dique (CARDIQUE, Colombia) under Resolution No. 0751 dated June 27, 2014.

Agave roots were air-dried, pulverized, and macerated using a 70:30 (v/v) hydroalcoholic mixture of ethanol absolute (Panreac AppliChem) and Milli-Q water as the solvent for the extraction of polar compounds, including phenolic acids and flavonoids. Extraction was carried out for 72 h, performing three consecutive cycles to maximize yield. The extracts obtained were combined and concentrated by vacuum evaporation using a rotary evaporator. The extract obtained was used to prepare a stock solution in dimethyl sulfoxide at a final concentration of 100 mg/mL. This stock solution was stored under refrigeration and protected from light until its use in subsequent analyses.

### Preparation of DMN arrays

The DMN arrays were made using negative polydimethylsiloxane (PDMS) molds, whose matrix contained a macrobasin that delimited a set of 100 microbasins arranged in a 10 × 10 array. Each microbasin had a height of 600 μm, a base size of 200 μm, and a pitch (distance between centers) of 500 μm, according to the specifications provided by SIGMA. To prepare the chitosan gel at a concentration of 4% w/v, 2 g of commercial chitosan powder (Chitosan low molecular weight, Sigma-Aldrich) was dissolved in 50 mL of 2% v/v acetic acid (Supelco). The mixture was subjected to ultrasonic treatment for 30 min at room temperature, obtaining a homogeneous gel with an initial pH of 4.274 (20.7°C), determined using Multiparameter equipment HI 2550 (Hanna Instruments). Subsequently, the pH was adjusted to 5.049 (18.3°C) by the controlled addition of 10 N sodium hydroxide (Supelco). Based on a preliminary pilot study in which four extract loading levels were initially tested in the microneedle formulations (0.25%, 0.50%, 1.0%, and 2.0% w/w). Loading levels ≥1.0% resulted in deformation, structural collapse, or inadequate demolding of the microneedle assemblies, even with prolonged drying time, and were therefore excluded from subsequent morphological and functional analyses. Non-loaded DMNs were prepared under identical conditions and used as negative controls. To form the arrays, 100 μL of the corresponding gel (chitosan gel or chitosan gel loaded with extract) was deposited in the macrobasin of the PDMS mold. The molds were then centrifuged at 3500 rpm for 30 min to ensure complete filling of the microbasins using a Heraeus Labofuge 400 Centrifuge. Subsequently, an additional amount of gel was added until the macrobasin was completely covered. The loaded molds were dried in an oven at 40 °C for 6 h to promote gel solidification. The DMN arrays were carefully separated from the molds and stored in resealable plastic bags until used in subsequent evaluations. The average weight of the DMN was determined from three units per concentration. Values of 6.5 ± 3.1 mg, 8.0 ± 2.6 mg, and 9.9 ± 2.7 mg were defined as the average weight of non-loaded DMN, 0.25% extract-loaded DMN, and 0.50% extract-loaded DMN, respectively.

Loaded and non-loaded DMN stock solutions were prepared with agave extract at a final concentration of 100 mg/mL, using dimethyl sulfoxide as the dissolution medium. Three microneedle arrays were used to prepare each stock solution, yielding a total of *n* = 9 loaded and *n* = 9 non-loaded DMN for analysis. These solutions were used to evaluate the *in vitro* biological potential of DMN. Once prepared, they were stored under refrigeration and in the dark until their use in subsequent analyses.

### Identification and quantification of bioactive compounds in the *A. tequilana* extract

To identify the key bioactive compounds underlying the functional properties of the extract, chemical profiling was performed using UHPLC-ESI-ORBITRAP-MS, following procedure established previously [84]. In brief, sample preparation involved dissolving the extract in a 1:1 (v/v) methanol-water mixture acidified with 0.2% formic acid, followed by vortex agitation for 5 min and sonication for 20 min to ensure thorough solubilization and homogeneity before analysis. Chromatographic separation was achieved on a Dionex Ultimate 3000 UHPLC platform (Thermo Scientific), featuring a binary gradient pump, autosampler, and temperature-controlled column compartment. A Hypersil GOLD Aq column (100 × 2.1 mm, 1.9 μm particle size) served as the stationary phase. The mobile phase comprised solvent A (water with 0.1% formic acid and 5 mM ammonium formate) and solvent B (methanol with 0.1% formic acid and 5 mM ammonium formate). A linear gradient elution was employed, transitioning from 100% A to 100% B over 8 min, maintaining 100% B for 4 min, followed by a 1-minute re-equilibration, totaling a 13-minute runtime. Mass spectrometric detection was performed in positive electrospray ionization mode (ESI+) with a capillary voltage set at 3.5 kV. Compound identification relied on full-scan acquisition with high mass accuracy (Δppm < 1), extraction of protonated molecular ions [M + H]^+^, isotopic pattern confirmation, and tandem MS fragmentation analysis. For ensuring precise and reliable measurement of target analytes, quantification was conducted using calibration curves constructed from certified reference standards: theobromine (Sigma-Aldrich, 99%), theophylline (Sigma-Aldrich, 99%), caffeine (Sigma-Aldrich, 99.9%), (-)-epigallocatechin gallate (EGCG) (PhytoLab, 99.5%), (-)-epicatechin (EC) (Sigma-Aldrich, 95.1%), (-)-epicatechin gallate (ECG) (PhytoLab, 98.5%), rutin (Sima-Aldrich, 97%), luteolin (Sigma-Aldrich, 98%), quercetin (Sigma-Aldrich, 98%), naringenin (Sigma-Aldrich, 98.6%), apigenin (AK Scientific LCMS, 99.2%), pinocembrin (Sigma-Aldrich, 95%), *p*-hydroxybenzoic acid (Sigma-Aldrich, 99.9%), Caffeic acid (Sigma-Aldrich, 99.5%), vanillic acid (STD-Aldrich, 97%), *p*-coumaric acid (Sigma-Aldrich, 98.6%), ferulic acid (Sigma-Aldrich, 99.6%), rosmarinic acid (Sigma-Aldrich, 97%), *trans*-cinnamic acid (Sigma-Aldrich, 99%), and ursolic acid (Sigma-Aldrich, 99.6%).

### Morphological and elemental analysis of raw materials

Scanning electron microscopy coupled to energy-dispersive X-ray spectroscopy (SEM-EDS) was employed for morphological and elemental analysis of commercial chitosan and agave extract. Initial morphological and qualitative elemental analysis was performed using a JEOL JSM-6490LV scanning electron microscope equipped with an Oxford Instruments INCA PentaFET-x3 EDX detector. These analyses were complemented by high-resolution imaging and detailed elemental mapping performed using a Thermo Fisher Scientific Apreo 2 S LoVac field emission scanning electron microscope (FESEM) equipped with an energy-dispersive X-ray (EDX) microprobe.

### Thermal characterization of raw materials and DMN arrays

The TGA measurements were run on Q500 equipment from TA instruments under a 50 mL/min nitrogen flow from 10 to 600 °C. The DSC measurements were run on Q200 equipment from TA instruments under a 50 mL/min nitrogen flow from 10 to 400 °C.

### SEM-based morphological and structural analysis of DMN arrays

The morphological and structural characterization of the DMN arrays was performed using FESEM with a ThermoFisher Scientific Apreo 2 microscope. In brief, the DMN arrays were mounted on aluminum supports using conductive adhesive tape and subsequently coated with a thin layer of gold via sputter coating to improve electrical conductivity and prevent charge accumulation during observation.

### Biological potential

The biological potential of agave extract was initially assessed using moderate concentrations to minimize selection bias and avoid concentration-related artifacts, allowing for an objective evaluation of skin-relevant activities under controlled *in vitro* conditions. Based on these preliminary findings, the extract was subsequently incorporated into DMN arrays to comparatively evaluate the preservation of selected bioactivities within the formulated systems. This evaluation was conducted exclusively between microneedles loaded with agave extract and non-loaded microneedles used as controls, without establishing direct comparisons with the agave extract or implying therapeutic applicability.

### Antioxidant capacity

For the antioxidant capacity assays, the extract stock solution was diluted 1:100 with Type II water, whereas the DMN stock solution was evaluated directly to ensure that the measurements remained within the linear range of the method.

For the FRAP assay, 10 μL of each diluted or undiluted sample were pipetted into individual wells of a 96‑well microplate. The FRAP working reagent was prepared by mixing 300 mM acetate buffer (pH 3.6), 20 mM FeCl₃·6H₂O, and 10 mM TPTZ (dissolved in 40 mM HCl) in volumetric ratios of 10:1:1. FRAP reagent (250 μL) was added to each well containing sample or Trolox standard (10 μL), and the microplate was incubated in the dark at 37 °C for 10 min to complete the redox reaction [85]. Absorbance was measured at 593 nm using a BioTek Synergy HT multimode microplate reader. A calibration curve was constructed with Trolox (6-hydroxy-2,5,7,8-tetramethylchroman-2-carboxylic acid, Sigma-Aldrich) standard to convert absorbances to μmol TE/100 g sample. The sensitivity and linearity of the assay were confirmed with correlation coefficients greater than 0.99. Each sample and standard were analyzed in triplicate. The mean ± standard deviation (SD) was calculated.

For the ABTS assay, the assay adapted from Bravo et al. [86] was used with minor modifications. The ABTS· + radical cation was generated by reacting 7 mM ABTS (2,2′-Azino-bis(3-ethylbenzothiazoline-6-sulfonic acid) diammonium salt, Sigma-Aldrich) with 2.5 mM potassium persulfate in PBS (phosphate-buffered saline, Sigma-Aldrich), followed by incubation in the dark at room temperature for 16 h to ensure complete radical formation. The oxidized solution was then diluted with PBS to obtain an absorbance of 0.70 ± 0.02 at 730 nm. In a 96-well microplate, 20 μL of Trolox standard or extract were mixed with 180 μL of the ABTS solution. After incubation in the dark for 15 min, the absorbance at 730 nm was recorded using a Synergy HT multimode reader. Antioxidant activity was calculated by interpolation with the Trolox calibration curve. Each sample and standard were analyzed in triplicate, and the mean ± SD was calculated.

The results were then expressed as TEAC (Trolox Equivalent Antioxidant Capacity), in µmol units of Trolox equivalent per 100 g of sample or DMN (µmol TE/100 g sample or DMN), calculated from a calibration curve obtained by linear regression, according to the Eq 1:


$$\begin{array}{c} \frac{\mu mol\;TE}{\text{100 }g\;sample}=\;\frac{\left(M*\;DF*\;V\right)}{m}*\text{100} \end{array}$$ (1)

where M is the molar concentration obtained from the calibration curve, DF the dilution factor applied to prepare the sample, V the volume in L in which the sample was initially prepared, and m the weight in g of the initial sample.

### Enzymatic inhibitory activity

The inhibition of tyrosinase, collagenase, and hyaluronidase by agave extract was evaluated at a concentration of 250 µg/mL, selected as a moderate level to assess its intrinsic biological activity and minimize potential concentration-related artifacts, including non-specific effects, signal saturation, or interference in enzyme assays.

For this purpose, the stock solution of agave extract was prepared by dissolving 25 mg of extract in 250 μL of dimethyl sulfoxide under vigorous stirring, resulting in a final concentration of 100 mg/mL. Conversely, the DMN stock solutions were prepared by dissolving three microneedle arrays in dimethyl sulfoxide to obtain an equivalent final concentration of 100 mg/mL.

The tyrosinase inhibition assay was performed using a spectrophotometric method reported from Quintero-Rincón et al. [87]. Briefly, 70 µL of sample solution or buffer control (prepared by diluting 2.1 µL of stock solution in 277.9 µL of 50 mM phosphate buffer, pH 6.5, to a concentration of 0.750 mg/mL) were added to wells of a 96-well microplate, followed by 30 µL of mushroom tyrosinase (333 units/mL). After a 5-minute incubation at room temperature, the reaction was initiated by adding 110 µL of 2 mM L-tyrosine. Kojic acid (Supelco, ≥ 99.0%) was used as positive control at a final concentration of 100 µM. Absorbance was measured at 480 nm every minute for 20 min using a BioTek Synergy HT multimode microplate reader (BioTek Instruments, Inc.). A decrease in absorbance compared to the control indicated tyrosinase inhibition by the extract. All measurements were performed in triplicate, and results were expressed as mean ± SD.

For the collagenase inhibition assay, the protocol of Barrantes & Guinea [88] was followed with minor modifications. Briefly, collagenase from *Clostridium histolyticum* (Sigma-Aldrich) was diluted in 50 mM tricine (Sigma-Aldrich) buffer (pH 7.5) with 10 mM calcium chloride (LOBA Chemie) and 400 mM sodium chloride (LOBA Chemie) to obtain a final concentration of 0.8 units/mL, ensuring the presence of Ca² ⁺ , essential for enzymatic activity. The FALGPA substrate (N-[3-(2-Furyl)acryloyl]-Leu-Gly-Pro-Ala, Sigma-Aldrich) was dissolved in the same buffer at 2 mM. For kinetic studies, serial dilutions between 0.5 and 2.5 mM were prepared. For evaluation, 5 µL of stock solution of extract was diluted in 495 µL of buffer. In addition, the control was prepared by diluting 5 µL of dimethyl sulfoxide in 495 µL of the same buffer. The experimental design was carried out in microplates, where 25 µL of control or inhibitor, 25 µL of enzyme and 50 µL of substrate were placed in each well. To correct the absorbance, additional wells were prepared with 25 µL of control or inhibitor and 75 µL of tricine buffer. After a 15-minute preincubation at 25 °C to stabilize enzyme activity, the substrate was added, and absorbance at 340 nm was recorded immediately and every 2 min for 20 min using a Multiskan SkyHigh Microplate Spectrophotometer (Thermo Scientific), monitoring substrate hydrolysis. The assay was performed in triplicate. EGCG (Sigma-Aldrich, 97%) was used as a positive control at a final concentration of 5 mM. The mean ± SD was calculated.

The hyaluronidase inhibition assay was conducted following the methodology of Liyanaarachchi et al. [89] with minor modifications. Briefly, a working solution was prepared by mixing 2.1 µL of the stock solution of extract with 18.9 µL of dimethyl sulfoxide in a 2 mL eppendorf tube, followed by the addition of 179 µL of type 1 water. The control solution was prepared by combining 21 µL of dimethyl sulfoxide with 179 µL of type 1 water. For enzyme mixtures, 40 µL of bovine testicular hyaluronidase type 1-S (4200 units/mL in 0.1 M acetate buffer, pH 3.5), Sigma-Aldrich, was combined with 100 µL of control or extract solution. These mixtures were incubated at 37 °C for 20 min to allow enzyme-inhibitor interaction. Hyaluronidase activation was then performed by adding 40 µL of 12.5 mM calcium chloride (LOBA Chemie) and incubating at 37 °C for 10 min. Subsequently, 100 µL of sodium hyaluronate (12 mg/mL in 0.1 M acetate buffer, pH 3.5), United States Pharmacopeia (USP) reference standard, was added and incubated at 37° for 40 min to permit substrate hydrolysis. The reaction was stopped by adding 20 µL of 0.9 M sodium hydroxide (Sigma-Aldrich) and 40 µL of 0.2 M sodium borate, followed by a 3-minute incubation in a boiling water bath to develop the colored compound. After cooling to room temperature, 100 µL of *p*-dimethylaminobenzaldehyde (LOBA Chemie) was added and incubated at 37 °C for 10 min to complete color development. Finally, 150 µL of each sample was transferred in triplicate to 96-well microplates, and absorbance was measured at 585 nm using a Multiskan SkyHigh Microplate Spectrophotometer (Thermo Scientific). EGCG (Sigma-Aldrich, 97%) was used as positive control at a final concentration of 10 mM. The mean ± SD was calculated.

For all three essays, the percentage of enzyme inhibition was calculated using Eq 2:


$$\begin{array}{c} Enzymatic\;inhibition\;(\%)=\;\frac{\left(A\;control-A\;sample\right)}{A\;control}*\text{100} \end{array}$$ (2)

where A control is the corrected absorbance of the reaction without inhibitor (negative control), and A sample is the corrected absorbance in presence of the DMN arrays or inhibitor.

### Cytotoxicity in HaCaT and A375 cell lines

Cytotoxicity was assessed by measuring cell viability in HaCaT (cat. no. 300493, CLS Cell Lines Service GmbH, Germany) and A375 (cat. no. 300110, CLS Cell Lines Service LLC, USA) cell lines after exposure to *A. tequilana* extract, prepared in a range of concentrations from a 100 mg/mL stock solution in dimethyl sulfoxide, and DMN array solutions (19.5–29.7 mg of DMN in culture medium). The MTT colorimetric assay, adapted from Caballero-Gallardo et al. [90] with minor modifications, was used. Cells cultured in Dulbecco’s Modified Eagle’s Medium (Sigma-Aldrich), supplemented with 10% fetal bovine serum (Sigma-Aldrich) and 1% penicillin/streptomycin (Sigma-Aldrich), were seeded in 96-well plates at a density of 1.5 × 10⁴ cells per well and incubated at 37 °C with 5% CO₂ until approximately 80% confluence was reached. The culture medium was subsequently replaced with 1:2 serial dilutions of the extract prepared in the same medium, at concentrations ranging from 3.9 to 500.0 μg/mL, followed by 24 h of incubation under the same conditions. After treatment, cells were washed with phosphate buffered saline (PBS, Sigma-Aldrich) and 50 μL of MTT reagent (thiazolyl blue tetrazolium bromide, Millipore) was added at a final concentration of 5 mg/mL. Cells were incubated for 3 h. Subsequently, the residual medium containing MTT was removed, and the formed formazan crystals were dissolved with 200 μL of dimethyl sulfoxide to measure the absorbance at 570 nm using a multimode microplate reader (Varioskan™ LUX, Thermo Fisher Scientific, Inc.). Cell viability percentages were normalized and calculated according to Eq 3, compared with the non-treated cells.


$$\begin{array}{c} Cell\;viability\;(\%)=\;\frac{Absorbance\;of\;the\;test\;}{Absorbance\;of\;control\;}\;x\;100 \end{array}$$ (3)

The normality of the data obtained in the cell viability assays was assessed using the Shapiro-Wilk test. To detect significant differences between experimental groups, a one-way analysis of variance (ANOVA) was used, followed by Dunnett’s test. The IC_50_ for each treatment was calculated by nonlinear sigmoid curve using a four-parameter logistic model, with 95% confidence intervals reported [87]. Statistical analyses and graphs of cell viability versus concentration were performed using GraphPad Prism 8.0 software. For the agave extract, experiments were performed in triplicate and independently repeated twice for HaCaT and A375 cells. In contrast, the experiments performed with the DMN arrays were carried out in triplicate and independently repeated twice for both HaCaT and A375 cell lines. Statistical significance was set at *p* < 0.05. Finally, the SI index was calculated using Eq 4:


$$\begin{array}{c} Selectivity\;index\;(SI)=\;\frac{IC50\;against\;normal\;cells}{IC50\;against\;target\;cells} \end{array}$$ (4)

Where, a SI greater than 1 indicates desirable selectivity of the extract for tumor cells or target cells and values greater than 3 indicate high selectivity [91,92].

## Supporting information

S1 Fig SEM micrographs and EDS iteration analysis of *A. tequilana* extract. (PDF)

S2 Fig SEM micrographs and EDS iteration analysis of commercial chitosan. (PDF)

S3 Fig TDA thermogram of commercial chitosan (A), *A. tequilana* extract (B), non-loaded DMN (C), and DMN loaded with extract: 0.25% *A. tequilana* extract (D) and 0.50% *A. tequilana* extract (E). (PDF)

S1 Table Results of the four iterations analyzed by EDS-SEM of *A. tequilana* extract. (PDF)

S2 Table Results of the four iterations analyzed by EDS-SEM commercial chitosan. (PDF)
