# Supplementary material for: Comprehensive chemical, morphological, thermal, and biological characterization of Agave tequilana extract and chitosan-based dissolving microneedle arrays
Source: PLoS One. 2026 Jun 5;21(6):e0350922. doi: 10.1371/journal.pone.0350922 (PMC13240934; doi:10.1371/journal.pone.0350922)
Supplement: S1 Fig — (PDF) [file pone.0350922.s001.pdf]

S1 Fig. SEM micrographs and EDS iteration analysis of *A. tequilana* extract.

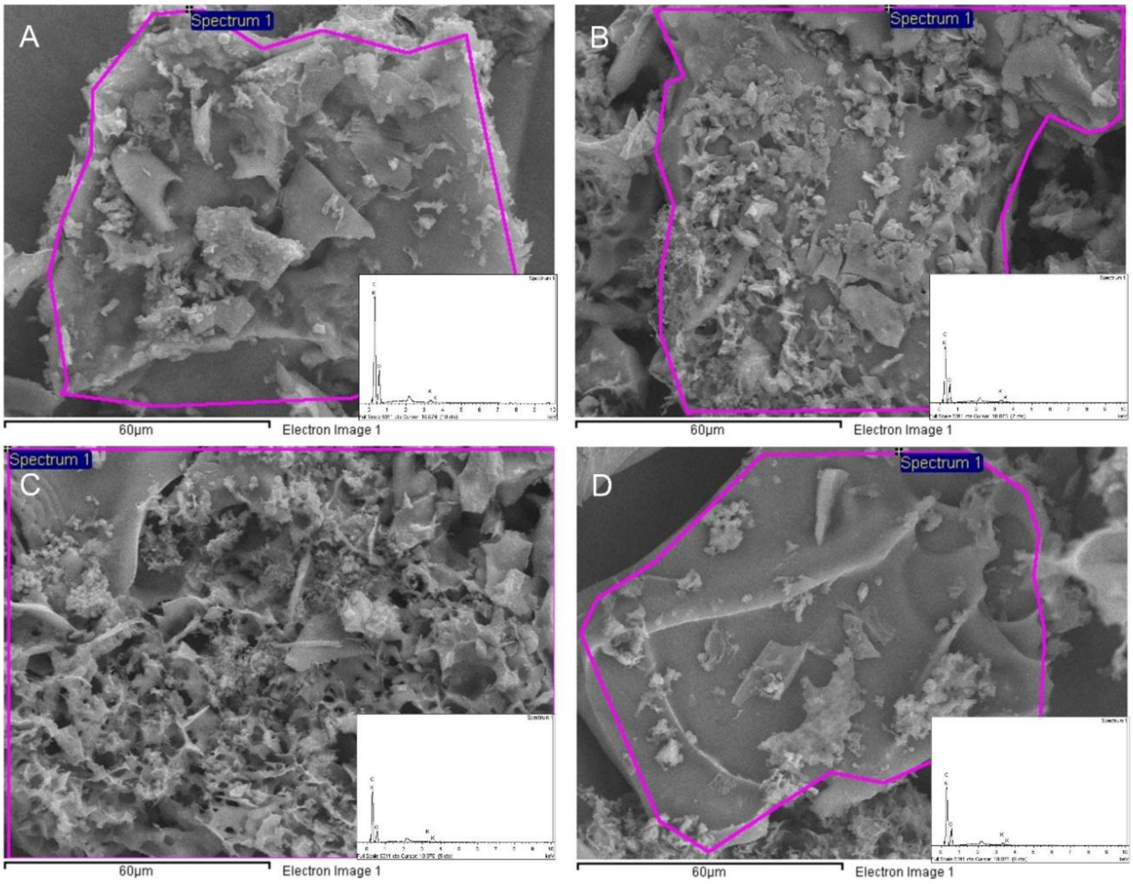

Scanning electron microscopy (SEM) images and energy-dispersive X-ray spectroscopy (EDS) spectra of the analyzed agave extract. Micrographs (A–D) show the surface morphology of the particles at a magnification scale where the bar corresponds to 60 µm. The regions selected for elemental analysis are highlighted in magenta. The insets correspond to the EDS spectra, where the x -axis represents energy (keV) and the y -axis represents counts (intensity). The peaks indicate the relative abundance of the detected elements. The most prominent signal corresponds to C (carbon), followed by minor contributions from O (oxygen) and K (potassium). Abbreviations: SEM, scanning electron microscopy; EDS, energy-dispersive spectroscopy; µm, micrometer; keV, kiloelectronvolt.
