# Supplementary material for: Comprehensive chemical, morphological, thermal, and biological characterization of Agave tequilana extract and chitosan-based dissolving microneedle arrays
Source: PLoS One. 2026 Jun 5;21(6):e0350922. doi: 10.1371/journal.pone.0350922 (PMC13240934; doi:10.1371/journal.pone.0350922)
Supplement: S2 Fig — (PDF) [file pone.0350922.s002.pdf]

S2 Fig. SEM micrographs and EDS iteration analysis of commercial chitosan.

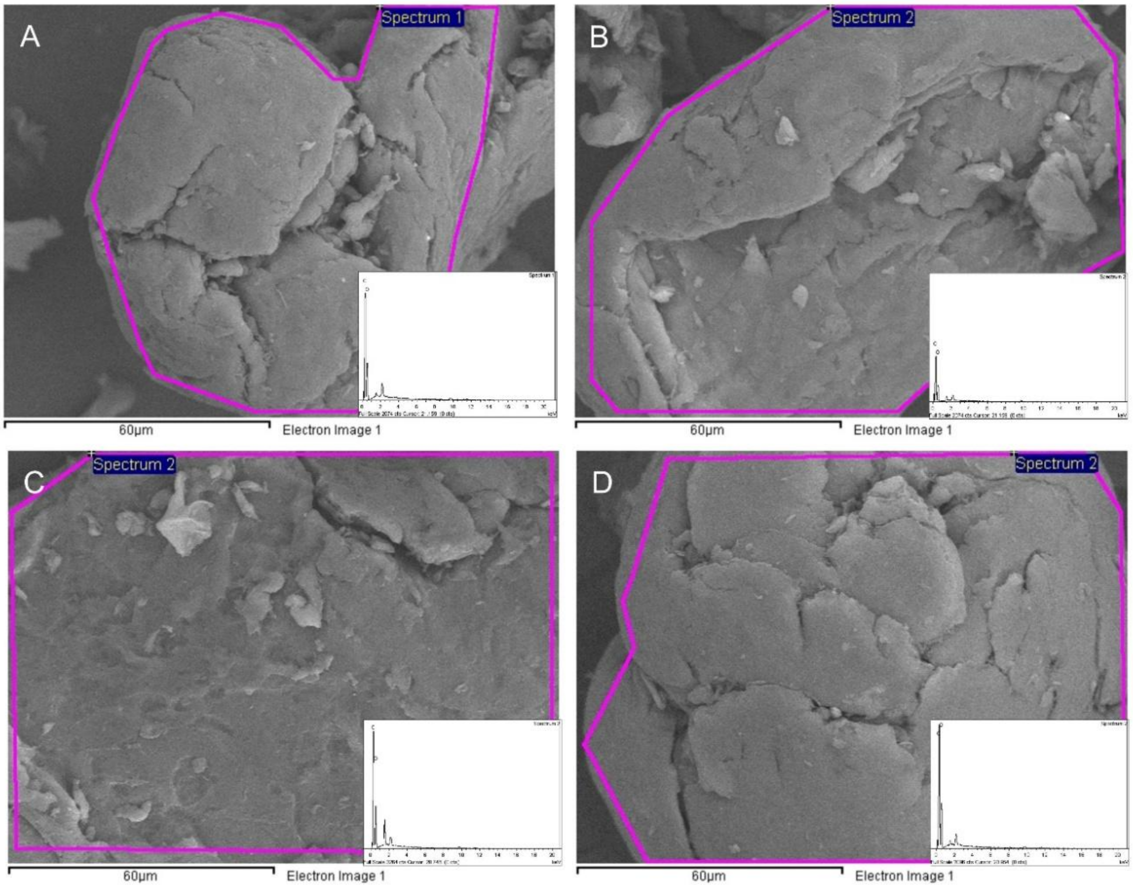

Scanning electron microscopy (SEM) images and energy-dispersive X-ray spectroscopy (EDS) spectra of the commercial chitosan sample. SEM micrographs (A–D) show the morphology of the particle surfaces at a scale where the bar represents 60 µm. The analyzed regions are highlighted in magenta and labeled as Spectrum 1 or Spectrum 2. The insets in each panel show the corresponding EDS spectra, where the x-axis represents energy (keV) and the y-axis represents counts (intensity). The most intense peak corresponds to C (carbon), with additional contributions from O (oxygen). Abbreviations: SEM, scanning electron microscopy; EDS, energy-dispersive spectroscopy; µm, micrometer; keV, kiloelectronvolt.
