# Supplementary material for: Comprehensive chemical, morphological, thermal, and biological characterization of Agave tequilana extract and chitosan-based dissolving microneedle arrays
Source: PLoS One. 2026 Jun 5;21(6):e0350922. doi: 10.1371/journal.pone.0350922 (PMC13240934; doi:10.1371/journal.pone.0350922)
Supplement: S3 Fig — (PDF) [file pone.0350922.s003.pdf]

S3 Fig. TDA thermogram of commercial chitosan (A), *A. tequilana* extract (B), non-loaded DMN (C), and DMN loaded with extract: 0.25% *A. tequilana* extract (D) and 0.50% *A. tequilana* extract (E).

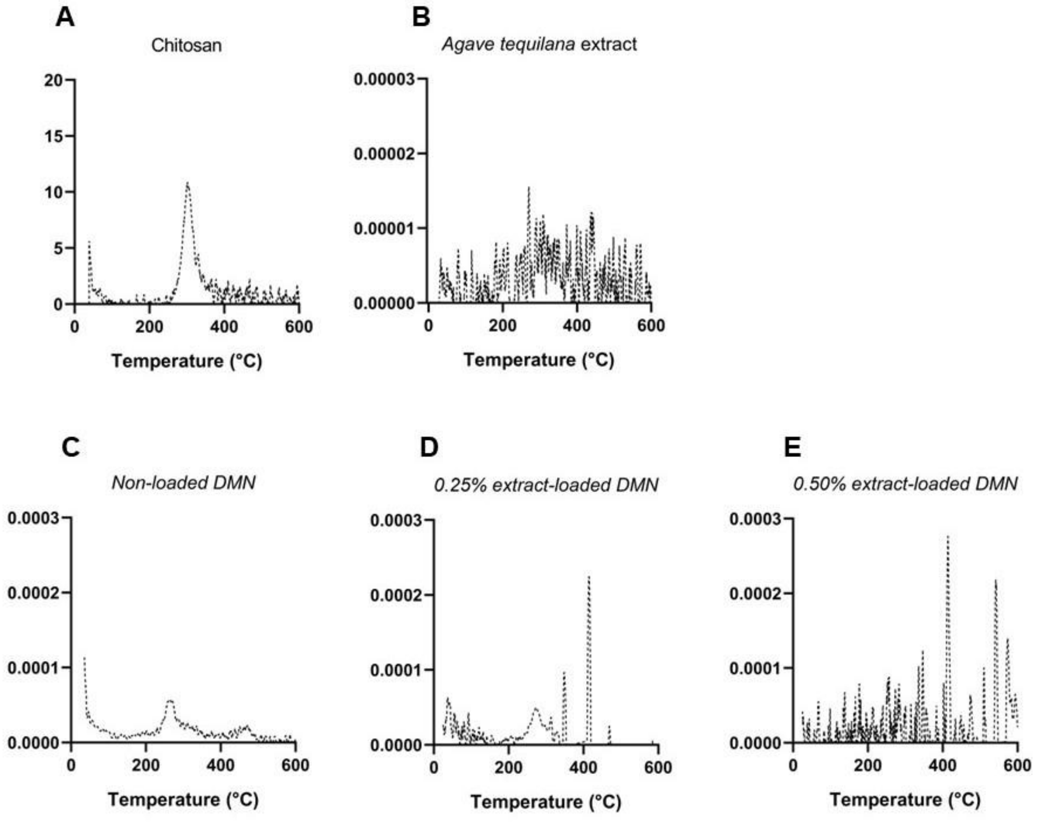

In the figure, the x-axis represents temperature (°C) in the range 0 to 600 °C, and the y-axis corresponds to the derived weight loss (dW/dT), expressed in arbitrary units. Each curve shows the thermal decomposition profile of the respective sample, with characteristic peaks indicating the degradation stages. Abbreviations: TDA, thermogravimetric analysis of derivatives; DMN, dissolving microneedles.
