# Supplementary material for: Comprehensive chemical, morphological, thermal, and biological characterization of Agave tequilana extract and chitosan-based dissolving microneedle arrays
Source: PLoS One. 2026 Jun 5;21(6):e0350922. doi: 10.1371/journal.pone.0350922 (PMC13240934; doi:10.1371/journal.pone.0350922)
Supplement: S1 Table — (PDF) [file pone.0350922.s004.pdf]

S1 Table. Results of the four iterations analyzed by EDS-SEM of *A. tequilana* extract.

**Number of Iterations**	**Element**	**App. Corrn.**	**Weight%**	**Weight% Sigma**	**Atomic%**
1	C	347.09	1.3852	63.35	0.70
	O	64.32	0.4489	36.23	0.70
	K	1.73	1.0430	0.42	0.05
2	C	284.81	1.3644	61.69	0.96
	O	57.99	0.4574	37.48	0.95
	K	2.94	1.0437	0.83	0.08
3	C	308.51	1.4581	68.10	1.04
	O	40.41	0.4139	31.43	1.04
	K	1.52	1.0431	0.47	0.09
4	C	301.97	1.3934	63.79	0.95
	O	53.54	0.4432	35.57	0.95
	K	2.26	1.0434	0.64	0.08
