# Supplementary material for: Comprehensive chemical, morphological, thermal, and biological characterization of Agave tequilana extract and chitosan-based dissolving microneedle arrays
Source: PLoS One. 2026 Jun 5;21(6):e0350922. doi: 10.1371/journal.pone.0350922 (PMC13240934; doi:10.1371/journal.pone.0350922)
Supplement: S2 Table — (PDF) [file pone.0350922.s005.pdf]

S2 Table. Results of the four iterations analyzed by EDS-SEM commercial chitosan.

Number of Iterations	Element	App. Corrn.	Weight%	Weight% Sigma	Atomic%
1	C	156.15	1.2887	56.55	0.98
	O	47.77	0.5132	43.45	0.98
2	C	145.17	1.2904	56.68	1.52
	O	43.99	0.5119	43.32	1.52
3	C	177.88	1.2911	56.74	0.74
	O	53.70	0.5114	43.26	0.74
4	C	180.54	1.2953	57.06	0.90
	O	53.31	0.5083	42.94	0.90
